# Supplementary material for: A simple model of the attentional blink and its modulation by mental training
Source: PLoS Comput Biol. 2022 Aug 29;18(8):e1010398. doi: 10.1371/journal.pcbi.1010398 (PMC9462776; doi:10.1371/journal.pcbi.1010398)
Supplement: S1 Text — (PDF) [file pcbi.1010398.s001.pdf]

S1 Text. Analytical approximation of blinking probability

In this mathematical appendix we derive a calculation of the blinking probability for a stimuli sequence with a sensory trace $\hat{y}(t)$, and an attentional load $y(t)$. We start by defining the following standard normal Gaussian process:

$$z(t) = \frac{y(t) - \hat{y}(t) * h_2(t) - \mu}{\sigma},$$

with stationary mental noise mean and standard deviation $(\mu(t), \sigma(t) = \mu, \sigma)$. A blinking event occurs when $y(t)$ reaches y_B, or equivalently, when $z(t)$ reaches $z_B(t)$, which is defined as follows:

$$z_B(t) = \frac{y_B - \hat{y}(t) * h_2(t) - \mu}{\sigma}.$$

Thus, the event of blink occurrence is the complementary event to $z(t)$ never reaching $z_B(t)$:

$$P(Blink) = 1 - P(\max_t z(t) < z_B(t)). \tag{S1}$$

To compute this numerically, we discretize the duration of each trial into into N equidistant samples $(t_1, ..., t_N)$. In our simulations, we used trial duration of 1.2 seconds, sampled every millisecond, giving $N = 1,200$ time samples. We now compute the discrete time blinking probability using the N standard normal i.i.d variables $(z(t_1), ..., z(t_N))$:

$$P(Blink) = 1 - P(\max_{i=1}^{N} z(t_i) < z_B(t_i)) = 1 - \prod_{i=1}^{N} P(z(t_i) < z_B(t_i))$$
$$= 1 - \prod_{i=1}^{N} \Phi(z_B(t_i)), \tag{S2}$$

where $\Phi(x)$ denotes the cumulative distribution function of the standard normal distribution. Since the product $\prod_{i=1}^{N} \Phi(z_B(t_i))$ is hard to evaluate exactly, we approximate it by omitting samples which are not near the peak of the attentional load (see Fig. 1 in the main text). These are samples for which $z_B(t_i) >> m$ where $m = \min_{i=1}^{N} z_B(t_i)$ and thus $\Phi(z_B(t_i)) \approx 1$, which does not have a substantial influence on the product $\Pi_i \Phi(z_B(t_i))$. This reflects the fact that blinking probability is negligible when the attentional load is low. We therefore approximate Eq. S2 as a product of n standard normal cumulative distributions evaluated at m, where n is the typical duration, in time samples, of the negative peak of $z_B(t)$:

$$P(Blink) \approx 1 - \prod_{i=1}^{n} \Phi(m) = 1 - P(M_n < m), \tag{S3}$$

with $M_n = \max_{i=1}^{n} z(t_i)$. The distribution of the maximum of Gaussian random variables is known to converge to the double exponential (or Gumbel) distribution, with the following asymptotic behavior [1]:

$$\lim_{n \to \infty} P(M_n < m) = e^{-e^{-a_n(m-b_n)}}, \tag{S4}$$

where $a_n = \sqrt{2 \ln n}$ and $b_n = a_n - \frac{\ln \ln n + \ln(4\pi)}{2\sqrt{2 \ln n}}$. Minimizing the squared difference between the simulated and analytical results, gave an optimal value of $n = 67$, closely approximating the simulated results (Fig. 2 in the main text).

References

[1] Gumbel EJ, Lieblein J. Some applications of extreme-value methods. The American Statistician. 1954;8(5):14–17.
