# Supplementary material for: A simple model of the attentional blink and its modulation by mental training
Source: PLoS Comput Biol. 2022 Aug 29;18(8):e1010398. doi: 10.1371/journal.pcbi.1010398 (PMC9462776; doi:10.1371/journal.pcbi.1010398)
Supplement: Attachment — Submitted filename: AB response letter.pdf [file pcbi.1010398.s002.pdf]

Response to reviewers - A simple model of attentional blink and its modulation by mental training

We thank the reviewers for their helpful comments. Please find the reviewers' comments reproduced below with our responses in red.

Reviewer #1

The authors present a dynamical-systems model of the AB, suggesting that meditation-induced modulations as previously observed in the pattern of attentional behavior as well as ERP responses are due to the interplay between attentional load and mental noise levels over time. In addition to demonstrating these effects in the context of the AB, the presented model can also account for lag-1 sparing and the spreading of lag-1 sparing.

General evaluation: The manuscript is well-written and presents an elegant model, accounting for several aspects of the AB phenomenon. The embedding with the existing literature is also done well. I lack the mathematical background to thoroughly judge the soundness of the model's approach, but the underlying mechanism is generally well explained. I found the model's surprising prediction that mental noise fluctuations would be increased following intense meditation of particular interest, but also found the 'clipping of the sensory trace' an intriguing and plausible mechanistic account of lag-1 sparing. I only have a couple of minor points as detailed below.

Minor points:

Fig 3: The clipping threshold is supposed to be represented by a solid red line, which is missing in the figure. I assume the solid black line was meant to be red?

We thank the reviewer for pointing out this discrepancy between the text and the figure - the clipping threshold is depicted as a solid black line and the blinking threshold as a dashed red line in all figures. We corrected the text to reflect this.

P9, line 220: please add 'amplitude' before 'reduction', so that it becomes clearer that the word 'reduction' refers to P3b amplitudes rather than a 'reduction in no-blink trials but not in blink trials'.

Done.

Fig 6 is rather small, the colors and crosses are rather hard to distinguish.

We enlarged Fig. 6.

P 10, line 250: finding -> findings

Fixed.

Figure 4: baseline activity in attentional load seems to be lower than in figure 3. Why?

Looking into this matter, we realized that the model does not provide a satisfactory explanation for the spreading of lag-1 sparing effect under the current parameter regime. Since this effect is somewhat disputed in the literature (see e.g. Chen & Zhou Atten Percept Psychophys (2015)) we preferred to remove this section altogether rather than try and fix the simulations. We thank the reviewer for pointing out this parameter discrepancy.

The authors write on page 11 that observed changes in behavior and P3b amplitude "can be explained as resulting from a combined reduction in the baseline and increase in the size of the fluctuations of the the mental noise". It would be useful to see what baseline is referred to here. Could it, along with the effects of meditation on the baseline, perhaps be visualized in a figure similar to Figures 1, 3 and 4?

Figure 7 was added to illustrate the effects of meditation on the attentional load baseline and variability and the resulting decrease in blinking probability.

P 11, line 273: the the mental noise -> the mental noise

Fixed.

Perhaps the title of the manuscript is literally a little too simple. A slightly less minimalistic title might better capture the attention of the reader and cover more of what the paper has to offer.

Changed the title to: A simple model of the attentional blink and its modulation by mental training

Reviewer #2:

Amir and colleagues proposed in their manuscript a single model of attention blink. Their model assumes that the blinking reflects an attentional capacity limitation due to T1 processing. Their model aimed to provide an account for the reduction of the AB effect following intensive mental training in the form of mindfulness meditation, with a corresponding reduction in T1-evoked P3b brain potentials. The concept of mental noise and its reduction by meditation training is central in the model.

The model is timely because there are still very little modelling efforts in the meditation literature. The strength of this work is that it reproduces with a parsimonious computational model both the behavioral performance, evoked brain potentials and training effect found in Slagter and colleagues. The modeling part and the simulations look adequate from what I can tell. The presentation of the literature and the results are satisfactory. The main limitation of this work is that it is not specific enough to model the regulatory mechanisms of mindfulness practices. This limitation somewhat reduces the explanatory power of this model. The model is ultimately about the role of mental noise on the AB rather than on the effects of mindfulness meditation on AB. The model does not explicitly model the top-down effects of meditation on the sensory trace. These limitations need to be highlighted and discussed in the text and in the manuscript in general.

More specifically,

While I agree with the idea that mindfulness meditation can lead to a reduction of mental noise (e.g. work on mind-wandering and meditation), meditation training might not necessary reduce mental noise and the reduction of mental noise might not be specific to meditation. More specifically, the meta-cognitive stance of mindfulness does not aim at blocking thoughts and emotions, but rather at becoming aware of them, to monitor them without rejecting them (e.g. see the literature on depression and mindfulness, e.g. Teasdale et al). So the goal of the practice is not directly to reduce mental noise but one's relationship to mental noise. Conversely, there are many activities which could reduce mental noise (walking in nature, being absorbed in some sportive active activity (i.e. concentrative exercise). These activities would not technically qualified as a mindfulness exercise. So this model of AB might not be helpful to understand the mechanism underlying Slagter et al.' findings.

In line with this idea, Van Vugt and Slagter reported in another study, a differential effect of two styles of meditation on the AB within the same participants: open monitoring style of mindfulness reduced more the AB than a focused style of mindfulness in expert meditators. The baseline level of mental noise during the performance cannot alone explain the behavioral differences found in these two meditative states (because the current model will behave similarly during each meditative state, and therefore I would think that this model can not explain the data from this other study).

This finding needs to be discussed in the discussion as a current limitation of the model.

Following this point, the current model posits that the regulation of the AB effect following the retreat is due to a reduction of mental noise, while Slater and colleagues interpreted their findings rather as produced by a cognitive stance modulating the engagement in the sensory trace itself in response to T1 stimulus. (here is the initial quote: "In this common style of meditation, one starts by focusing or stabilizing concentration on an object such as the breath. Then one broadens one's focus, cultivating a non-reactive form of sensory awareness or ''bare'' attention. This form of attention is non-reactive in the sense that, ideally one does not become caught up in judgments and affective responses about sensory or mental stimuli.".

The authors need to state more clearly to the readers that their model is not meant to model the regulatory effect of mindfulness as proposed by Slagter et al. In the current formulation, the model cannot account for a top-down influence on the sensory trace specific to the meditation. It is rather a task unrelated lack of top-down influence (i.e. the mental noise), which penalizes the magnitude of the P3b (Mental noise fluctuations scales the subject-specific P3b amplitudes line 136).

We added the following two paragraph to the discussion, addressing these points:

The goal of mindfulness meditation is often described as becoming aware of ongoing mental activity and changing one's attitude towards it, rather than actively manipulating it \cite{teasdale1999metacognition}. Indeed, Slagter et al. interpreted the effects of meditation on the attentional blink as a top-down regulation of the engagement with the sensory trace in response to the first target \cite{Slagter2007}. In its current formulation, our model does not account for such meditation-specific effects on the sensory trace. Rather, it implements a

task-unrelated modulation of mental noise in the attentional processing stage which modulates behavioral performance and P3b amplitudes. Admittedly, while mindfulness meditation may reduce mental noise, it does not necessarily do so. Conversely, mental noise can be reduced in other ways as well. The model predicts that changes in mental noise would affect attentional performance and P3b amplitudes in a similar way to meditation.

In a more recent study, van Vugt et al. compared attentional blink performance in expert meditators performing two different types of meditation: \emph{focused attention}, in which attention is focused tightly on an object, and \emph{open monitoring}, in which one is simply aware of whatever comes into awareness \cite{VanVugt2014}. This study reported a smaller attentional blink during open monitoring compared to focused attention meditation, indicating that particular meditative states can also influence the magnitude of the attentional blink. While that finding was also interpreted in terms of reduced top-down processing of T1, our model provides an alternative interpretation, namely that open monitoring meditation causes a larger reduction in mental noise compared to focused attention meditation.

I was surprised that the authors did not model explicitly the non-target visual stimuli (visual letters in Slagter et al). They suggested at the end of the text that it could be added. It would have made their model more physiologically plausible as the P3b is often viewed as reflecting context-updating (Polich, 2003) (without visual letter there are no context updating..). In this view, stimuli enter the processing system and a memory comparison process is engaged that ascertains whether the current stimulus is either the same as the previous stimulus or not. If the incoming stimulus is not the same and the subject allocates attentional resources to the target, the neural representation of the stimulus environment is changed or updated, such that a P300 (P3b) potential is generated in addition to the sensory evoked potentials. I would encourage the authors to do it, but it is not a requirement. At least the authors need to discuss whether they think their second stage model is neurophysiological plausible or whether it is just described the slow time scale of attentional processes.

Addressing the comment about the role of distractors, we added the following paragraph at the end of the discussion:

The role of distractors in generating the attentional blink is a matter of ongoing debate. Under certain accounts, the attentional blink is caused by the appearance of a distractor after T1, triggering a disruption in attentional control \cite{Olivers2008,DiLollo2005}. These accounts are in line with the view of the P3b potential as reflecting context updating \cite{Polich2007}, in this case switching from distractor to target processing. Another family of accounts, attributes the attentional blink to resource capacity limitations cite{Chun1995,jolicoeur1998}. Our model supports such capacity limitation accounts, suggesting that attentional resource consumption is determined by the interstimulus interval. Targets are assumed to be identified and distinguished from distractors at the pre-attentional sensory stage and therefore distractors do not draw any

attentional resources. Under this view, P3b amplitudes reflect attentional resource allocation rather than a context update.

Addressing the comment about neurophysiological plausibility, we added the following text to the first paragraph in the discussion:

The focus of the model on explaining the interaction between mental noise and the timescale of attentional processing, rather than providing a neurophysiologically plausible substrate for attentional resource allocation.

Sincerely,

Israel Nelken and Nadav Amir
